# Single-pool model urea clearance index is associated with sarcopenia and nutritional status in patients undergoing maintenance hemodialysis: a cross-sectional study

**DOI:** 10.1186/s12882-024-03510-4

**Published:** 2024-03-05

**Authors:** Yan Li, Tingting Xing, Rong Xu, Yan Liu, Xiaoshi Zhong, Yun Liu, Rongshao Tan

**Affiliations:** 1 Department of Nephrology, Guangzhou Red Cross Hospital of Jinan University, Guangzhou, China; 2 Department of Clinical Nutrition, Institute of Disease-Oriented Nutritional Research, Guangzhou Red Cross Hospital of Jinan University, Guangzhou, China

**Keywords:** Maintenance hemodialysis, Nutritional status, Sarcopenia, Single-pool Kt/V_urea_

## Abstract

**Background:**

The single-pool model urea clearance index (single-pool Kt/V_urea_; spKt/V) is the most commonly used method for dialysis adequacy assessment. However, only a few studies have examined the relationship between spKt/V values and parameters related to sarcopenia and nutritional status. This study aimed to evaluate whether the spKt/V is an indicator of sarcopenia and nutritional status in patients undergoing maintenance hemodialysis (MHD).

**Methods:**

A total of 142 patients were included in this single-center, cross-sectional study. Venous blood samples were collected shortly before the hemodialysis session. The adequacy of dialysis in patients receiving MHD was assessed using spKt/V. Sarcopenia was identified according to the Asian Working Group for Sarcopenia (2019) definition. Receiver operating characteristic curve and area under the curve were used to evaluate the predictive value of spKt/V in sarcopenia. Univariate and multivariate binary logistic regression analyses were used to determine the association between spKt/V and sarcopenia and nutritional status.

**Results:**

The mean spKt/V level was 1.3 ± 0.2, the prevalence of sarcopenia was 15.5% in patients on MHD. The best cutoff value of spKt/V in sarcopenia was 1.45 for both sexes, 1.33 and 1.45 for men and women, respectively (*P* < 0.05). The multivariate binary logistic regression shown that the spKt/V was independently positively associated with sarcopenia (OR = 122.88, 95% CI = 0.64–0.87, *P* = 0.002). Grouping spKt/V by the best cutoff value, when spKt/V ≥ 1.45, the OR of sarcopenia was 11.75 (95% CI = 3.16–43.67, *P* < 0.001). Subgroup analyses showed that when spKt/V ≥ 1.33 in men and spKt/V ≥ 1.45 in woman, the OR of sarcopenia was 9.73 (95% CI = 2.25–42.11, *P* = 0.002) and 14.52 (95% CI = 1.06–199.67, *P* = 0.045), respectively.

**Conclusions:**

The present study showed that spKt/V was an important influencing factor of sarcopenia and malnutrition in Asian patients on MHD.

**Supplementary Information:**

The online version contains supplementary material available at 10.1186/s12882-024-03510-4.

## Introduction

Patients with end-stage kidney disease (ESKD) on maintenance hemodialysis (MHD) often experience various metabolic disorders, among which sarcopenia is a serious complication characterized by muscle protein loss, decreased muscle strength, and impaired functionality. Sarcopenia in patients on dialysis increases the risk of cardiovascular events, infections, and all-cause mortality [1] while imposing a strain on healthcare insurance systems. Patients with CKD are prone to malnutrition or protein-energy wasting (PEW), which places them at risk of poor prognoses [2]. The worldwide prevalence of PEW ranges from 11 to 54% in patients with CKD stages 3–5 [3] and 28–54% in those undergoing dialysis [4].

Currently, the single-pool model urea clearance index (urea clearance multiply by dialysis session duration/volume of urea distribution [V_urea_]; spKt/V) is the most commonly used method for dialysis doses assessment [5]. According to the National Kidney Foundation-Kidney Disease Outcomes Quality Initiative (NKF-KDOQI) Clinical Practice Guidelines, the recommended minimum level for patients with little or no residual renal function (RKF) is set at 1.2 spKt/V (equal to 65% urea reduction rate [URR]) [6]. Studies indicate that Vurea can serve as an indicator of skeletal muscle mass and nutritional health. Patients with low muscle mass or malnutrition typically have higher Kt/V values, which ultimately leads to poor prognosis [7]. Moreover, spKt/V values ≥ 1.4 are negatively correlates with albumin, body mass index (BMI) and Geriatric Nutrition Risk Index (GNRI) in patients on hemodialysis [8]. These findings demonstrate that dialysis dose is intricately linked to skeletal muscle and nutritional status. Interpretation of the available data on the relationship between Kt/V and patient survival suggests that increasing the dialysis dose appears to be beneficial to the patient, but reaching the “overdialysis” threshold may lead to higher mortality [9]; hence, the optimal threshold dose of dialysis is still debatable.

To our knowledge, studies have not utilized the spKt/V to diagnose and evaluate sarcopenia in patients on MHD, and only a few quantitative studies examined the relationship between spKt/V values and parameters related to sarcopenia and nutritional status. Therefore, this cross-sectional study aimed to examine the relationship between spKt/V and sarcopenia and nutritional status in patients on MHD.

## Methods and materials

### Population and study design

Patients who regularly received MHD at our hemodialysis center in September 2022 were enrolled in this single-center, cross-sectional study. The inclusion criteria were: (1) performed maintenance hemodialysis regularly for more than 3 months, 3 times a week, and for four hours each time, (2) aged ≥ 18 years, (3) treatment with bicarbonate dialysate and polysulfone membranes, and (4) provision of informed consent. The non-inclusion criteria were: (1) patients with pacemakers; (2) those with comorbid malignant tumors, decompensated cirrhosis, heart failure, acute myocardial infarction, cerebrovascular accident, or severe infections within 3 months; or (3) those with physical disabilities or who could not cooperate with the study for other reasons. This study adhered to the principles of the Declaration of Helsinki and was approved by the appropriate ethics committee (No. 2023-040-01). Venous blood samples were collected shortly before the hemodialysis session, and demographic, clinical, biochemical, and body composition measurement data were recorded.

### Demographic, clinical, and laboratory parameters

Data on the history of diabetes, sex, age, and dialysis vintage were collected. The blood samples obtained before dialysis were analyzed at the Clinical Laboratory Department of our hospital using biochemical tests. Subsequently, serum creatinine, serum albumin, proalbumin, and highly sensitive C-reactive protein. According to the modification of diet in renal disease (MDRD) to calculate the estimated glomerularfiltration rate (eGFR) [10]:

$$\begin{array}{l}\text{e}\text{G}\text{F}\text{R} \left(\text{m}\text{L}\,\text{m}\text{i}\text{n} {-}^{1} 1.73 \text{m}{-}^{2}\right)\\= 186 \times \text{s}\text{e}\text{r}\text{u}\text{m}\,\text{c}\text{r}\text{e}\text{a}\text{t}\text{i}\text{n}\text{i}\text{n}\text{e} \left(\text{m}\text{g} \text{d}\text{L}{-}^{1}\right) {-}^{1.154}\\\times \left[\text{a}\text{g}\text{e} \right(\text{y}\text{e}\text{a}\text{r}\text{s}\left)\right]{-}^{0.203} \times \left(0.742 \text{i}\text{f}\,\text{f}\text{e}\text{m}\text{a}\text{l}\text{e}\right) \times \left(1.21 \text{i}\text{f}\,\text{b}\text{l}\text{a}\text{c}\text{k}\right)\end{array}$$

### Assessment of dialysis adequacy

The adequacy of dialysis in patients receiving MHD was assessed using spKt/V and URR. The spKt/V was calculated according to second-generation logarithmic estimates of spKt/V:

$$ \text{sp}\text{K}\text{t}/\text{V} = -\text{l}\text{n} (\text{R} - 0.008 \times \text{t}) + (4 - 3.5 \times \text{R}) \times \text{U}\text{F}/\text{W}$$

Where R is the ratio of pre- to posthemodialysis concentrations of BUN, t is the dialysis session duration (in hours), UF is the amount of ultrafiltration (L) during the given hemodialysis session, and W is the post-hemodialysis weight (kg) [11]. Pre- and posthemodialysis urea levels were obtained using the slow-flow technique described in the NKF-KDOQI guidelines [6]. The URR was calculated according to the following formula [12]:

$$\begin{array}{l}\text{U}\text{R}\text{R}= \text{p}\text{r}\text{e}\text{h}\text{e}\text{m}\text{o}\text{d}\text{i}\text{a}\text{l}\text{y}\text{s}\text{i}\text{s}\,\text{B}\text{U}\text{N}\\-\text{p}\text{o}\text{s}\text{t}\text{h}\text{e}\text{m}\text{o}\text{d}\text{i}\text{a}\text{l}\text{y}\text{s}\text{i}\text{s}\,\text{B}\text{U}\text{N}/\text{p}\text{r}\text{e}\text{h}\text{e}\text{m}\text{o}\text{d}\text{i}\text{a}\text{l}\text{y}\text{s}\text{i}\text{s}\,\text{B}\text{U}\text{N}\end{array}$$

### Pinch strength and grip strength measurement

Pinch strength and grip strength were measured using the BASELINE digital Pinch Force Tester (12–0081, Fabrication Enterprises Inc., USA) and the BASELINE digital Grip Force Tester (12–0091, Fabrication Enterprises Inc., USA). The pinch force tester and grip force tester were placed in the hand of MHD patents without a fistulae or the dominant hand before dialysis. Participants were asked to apply as much pinch and grip strength as possible to the instrument. The measurements were repeated three times and their maximum values were taken.

### Body composition measurement

The patients’ body composition was measured using a body composition analyzer (Multiscan 5000; Bodystat, Isle of Man, UK) by bioimpedance spectroscopy analysis (BIS). Measurements were performed with patients in the supine position, with electrode sheets attached to the hands and feet on the side without a fistula for hemodialysis, and the measurements were performed after inputting the relevant information. Lean body mass, phase angle (PhA), body cell mass, extracellular water (ECW), intracellular water (ICW), and the ECW/ICW ratio were measured. The equations for calculating appendicular skeletal muscle (ASM) and appendicular skeletal muscle index (ASMI) are as follows:

$$\begin{array}{l}\text{A}\text{S}\text{M} = (0.6974 \times (\text{h}\text{e}\text{i}\text{g}\text{h}\text{t} \times 100\left)^2 \right)/\text{Z}50) + (-55.24 (\text{Z}250/\text{Z}5))+\\(-10,940\times (1/\text{Z}50))+ 51.33\end{array}$$

for men; and

$$\begin{array}{l}\text{A}\text{S}\text{M}=(0.6144\times (\text{h}\text{e}\text{i}\text{g}\text{h}\text{t}\times 100\left)^{2}\right)/\text{Z}50) + (-36.61(\text{Z}250/\text{Z}5))+\\(-9322\times (1/\text{Z}50)) + 37.91\end{array}$$

for women.

Where Z is the reactance at different frequencies in Ω, height in m, and ASM in kg; and ASMI was calculated as ASM/height^2^ (kg/m^2^) [13]. BMI was calculated as weight/height^2^ (kg/m^2^).

### Assessment of Sarcopenia

Sarcopenia was defined in accordance with the criteria of the 2019 Asian Sarcopenia Working Group on Sarcopenia: (1) ASMI < 7.0 kg/m^2^ for men and ASMI < 5.7 kg/m^2^ for women; (2) Grip strength < 28 kg for men and < 18 kg for women [14].

### Calculation of nutritional indices

The GNRI was calculated according to the following formula:

$$\begin{array}{l}\text{G}\text{N}\text{R}\text{I} = 1.489 \times \text{s}\text{e}\text{r}\text{u}\text{m}\,\text{a}\text{l}\text{b}\text{u}\text{m}\text{i}\text{n} (\text{g}/\text{L})\\+ 41.7 \times (\text{a}\text{c}\text{t}\text{u}\text{a}\text{l}\,\text{b}\text{o}\text{d}\text{y}\,\text{w}\text{e}\text{i}\text{g}\text{h}\text{t}/\text{i}\text{d}\text{e}\text{a}\text{l}\,\text{b}\text{o}\text{d}\text{y}\,\text{w}\text{e}\text{i}\text{g}\text{h}\text{t})\end{array}$$

Where ideal body weight was calculated as 22 (kg/m^2^) × height. If the actual body weight was greater than the ideal body weight, the value of “(actual body weight/ideal body weight)” was set to 1 [15]. Currently, several studies have shown that a GNRI < 91.2 in patients on MHD can be defined as a risk of malnutrition [16]. This definition was adapted in the present study.

The modified Creatinine Index (mCI) was calculated according to the following formula [17]:

$$\begin{array}{l}\text{m}\text{C}\text{I}\,(\text{m}\text{g}/\text{k}\text{g}/\text{d}\text{a}\text{y}) = 16.21 + 1.12 \times (0\,\text{f}\text{o}\text{r}\,\text{w}\text{o}\text{m}\text{e}\text{n};\,1\,\text{f}\text{o}\text{r}\,\text{m}\text{e}\text{n})\,-\\0.06 \times \text{a}\text{g}\text{e} \left(\text{y}\text{e}\text{a}\text{r}\text{s}\right)- 0.08 \times \text{s}\text{p}\text{K}\text{t}/\text{V}\,\text{f}\text{o}\text{r}\,\text{u}\text{r}\text{e}\text{a}+ 0.009 \times \\\text{p}\text{r}\text{e}-\text{h}\text{e}\text{m}\text{o}\text{d}\text{i}\text{a}\text{l}\text{y}\text{s}\text{i}\text{s}\,\text{c}\text{r}\text{e}\text{a}\text{t}\text{i}\text{n}\text{i}\text{n}\text{e}\,({\mu }\text{m}\text{o}\text{l}/\text{L})\end{array}$$

### Statistical analyses

SPSS (version 25.0; IBM Corp, Armonk, NY, USA) and R software (version 4.2.1) was used for statistical analyses. Continuous variables with a normal distribution were described as mean ± standard deviation, and those with non-normal distribution as median (interquartile range [IQR]). Categorical variables were described using percentages. Participants were divided into non-sarcopenic and sarcopenic groups. Comparisons between two groups were performed by two independent-samples *t* test, χ^2^ test, or Mann-Whitney U test. Receiver operating characteristic (ROC) curve and its respective area under the curve (AUC) were used to evaluate the predictive value of spKt/V in sarcopenia and GNRI. The cutoff value was defined as the maximum value of (sensitivity - [1-specificity]). Univariate analyses were conducted to verify the correlation between spKt/V and sarcopenia parameters and nutritional parameters. In multivariate binary logistic regression analyses, potentially relevant variables or known to be important in the physiology of sarcopenia: sex, age, diabetes, dialysis vintage and BMI, were included to determine whether the spKt/V and the best cutoff value of spKt/V values for both sexes, men and women was independently associated with sarcopenia. A two-tailed *P*-value < 0.05 was considered statistically significant.

## Results

### General clinical characteristics of the participants

**Fig. 1:**
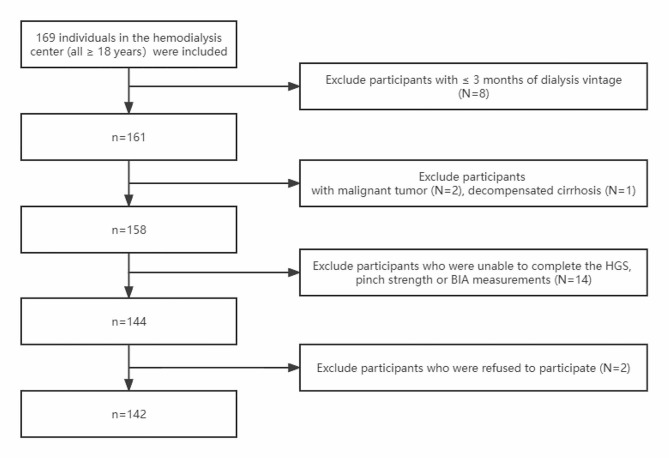
Sample selection flowchart of the study

The screening process is shown in Fig. 1. We reported in Table 1 the characteristics of the overall population as well as the comparison between Non-sarcopenia and Sarcopenia groups. The partial indicators of muscle strength, body composition, and nutritional indices were significantly lower in the sarcopenia group compared to the non-sarcopenia group. The age, spKt/V in the sarcopenia group were significantly higher than those of the non-sarcopenia group (*P* < 0.05).

**Table 1:** Baseline characteristics of the participants

Characteristic	Total (*n* = 142)	Non-sarcopenia (*n* = 120)	Sarcopenia (*n* = 22)	*p*
Age, years	66.3 ± 12.6	65.2 ± 12.2	72.2 ± 13.1	0.016
Male, n (%)	92 (64.8)	78 (65)	14 (63.6)	0.902
Dialysis vintage, months	41.9 ± 33.2	42.2 ± 34.1	40.2 ± 28.4	0.797
Diabetes mellitus, n (%)	79 (55.6)	72 (60)	7 (31.8)	0.014
Creatinine, µmol/L	926.7 ± 283.1	938.2 ± 295.4	863.6 ± 196.9	0.257
eGFR, ml/min/1.73 m^2^	5.0 ± 4.1	5.1 ± 4.5	4.6 ± 1.2	0.618
spKt/V	1.3 ± 0.2	1.3 ± 0.2	1.5 ± 0.2	< 0.001
URR	0.7 ± 0.1	0.7 ± 0.1	0.7 ± 0.1	< 0.001
Albumin, g/L	38.6 ± 4.1	38.9 ± 4.2	36.9 ± 3.6	0.042
Prealbumin, mg/L	310.0 ± 67.8	313.8 ± 67.0	289.2 ± 70.3	0.117
hs-CRP, mg/L	10.2 ± 11.5	9.5 ± 10.0	14.4 ± 17.5	0.064
Grip strength, kg	17.6 ± 8.7	18.5 ± 8.8	12.5 ± 5.3	0.002
Pinch strength, kg	5.3 ± 2.2	5.5 ± 2.3	4.5 ± 1.4	0.061
Lean, kg	46.9 ± 11.3	48.9 ± 10.5	35.5 ± 8.0	< 0.001
PhA, °	4.4 ± 1.6	4.6 ± 1.6	3.4 ± 0.8	< 0.001
body cell mass, kg	25.1 ± 9.0	26.2 ± 8.9	17.4 ± 5.4	< 0.001
ECW, Lt	18.5 ± 3.7	15.9 ± 2.8	14.1 ± 2.7	< 0.001
ICW, Lt	19.8 ± 4.9	18.5 ± 7.5	14.0 ± 4.2	< 0.001
ECW/ICW	1.0 ± 0.2	0.9 ± 0.2	1.1 ± 0.2	< 0.001
BMI, kg/m^2^	23.1 ± 3.4	23.4 ± 3.4	21.4 ± 2.7	0.012
ASM, kg	22.1 ± 6.7	23.4 ± 6.4	15.1 ± 2.8	< 0.001
GNRI, score	97.5 ± 6.7	98.2 ± 6.6	94.0 ± 6.5	0.006
mCI, mg/kg/day	21.2 ± 3.2	21.4 ± 3.3	20.2 ± 2.5	0.128

spKt/V, single-pool Kt/V_urea_; URR, urea reduction ratio; eGFR, estimated glomerular filtration rate; hs-CRP, serum hyper-sensitive C-reactive protein; PhA, phase angle; ICW, intracellular water; ECW, extracellular water; ECW/ICW, extracellular to intracellular water ratio; BMI, body mass index; ASM, appendicular skeletal muscle mass; GNRI, Geriatric Nutritional Risk Index; mCI, modified creatinine index

Continuous variables are expressed as mean ± standard deviation or median with interquartile range in case of nonnormally distributed data, and categorical variables are expressed as percentages

### Correlations between spKt/V and sarcopenia parameters and nutritional parameters

The results of univariate binary logistic regression analysis are shown in Table 2. We found that much of the lower levels indicators of muscle strength, body composition, and nutritional indices were related to spKt/V (*P* < 0.05).

**Table 2:** spKt/V and sarcopenia parameters and nutritional parameters

Characteristic	OR	95% CI	*p*
Age, years	1.02	0.99–1.05	0.189
Male, n (%)	6.56	3.01–14.29	< 0.001
Dialysis vintage, months	1.01	1-1.02	0.119
Diabetes mellitus, n (%)	0.77	0.38–1.56	0.46
Albumin, g/L	0.99	0.91–1.08	0.808
Prealbumin, mg/L	1	1-1.01	0.61
hs-CRP, mg/L	0.99	0.96–1.03	0.674
Grip strength, kg	0.93	0.89–0.98	0.004
Pinch strength, kg	0.72	0.58–0.88	0.001
Lean, kg	0.89	0.85–0.93	< 0.001
PhA, °	0.84	0.62–1.14	0.265
body cell mass, kg	0.9	0.84–0.96	0.001
ECW, Lt	0.73	0.63–0.84	< 0.001
ICW, Lt	0.77	0.69–0.87	< 0.001
ECW/ICW	8.56	1.57–46.73	0.013
BMI, kg/m^2^	0.87	0.77–0.98	0.023
ASMI, kg/m^2^	0.52	0.39–0.7	< 0.001
GNRI < 91.2, score	2.41	0.84–6.89	0.102
mCI, mg/kg/day	0.86	0.76–0.97	0.012
Sarcopenia, n (%)	5.02	1.92–13.12	0.001

OR, odds ratio; CI, confidence interval; spKt/V, single-pool Kt/V_urea_; hs-CRP, serum hyper-sensitive C-reactive protein; PhA, phase angle; ICW, intracellular water; ECW, extracellular water; ECW/ICW, extracellular to intracellular water ratio; BMI, body mass index; ASMI, appendicular skeletal muscle mass index; GNRI, Geriatric Nutritional Risk Index; mCI, modified creatinine index

### spKt/V in Sarcopenia and GNRI diagnosis

**Fig. 2:**
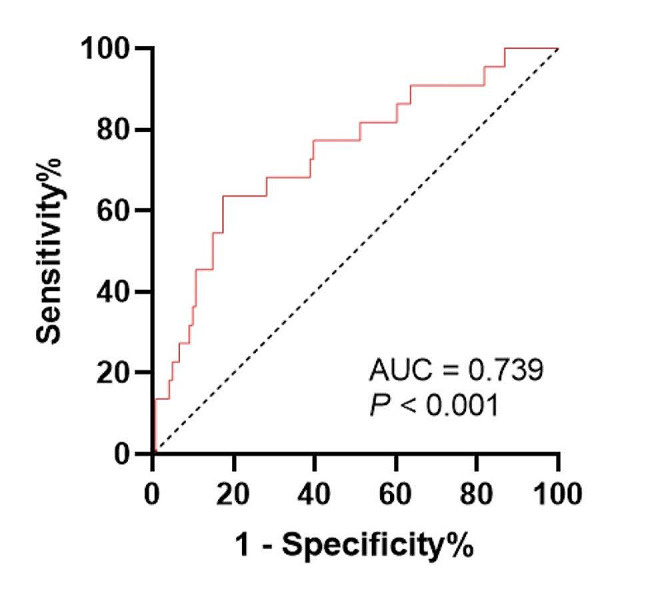
Receiver operating characteristic curve assessing the optimal thresholds of as a marker for sarcopenia

**Fig. 3:**
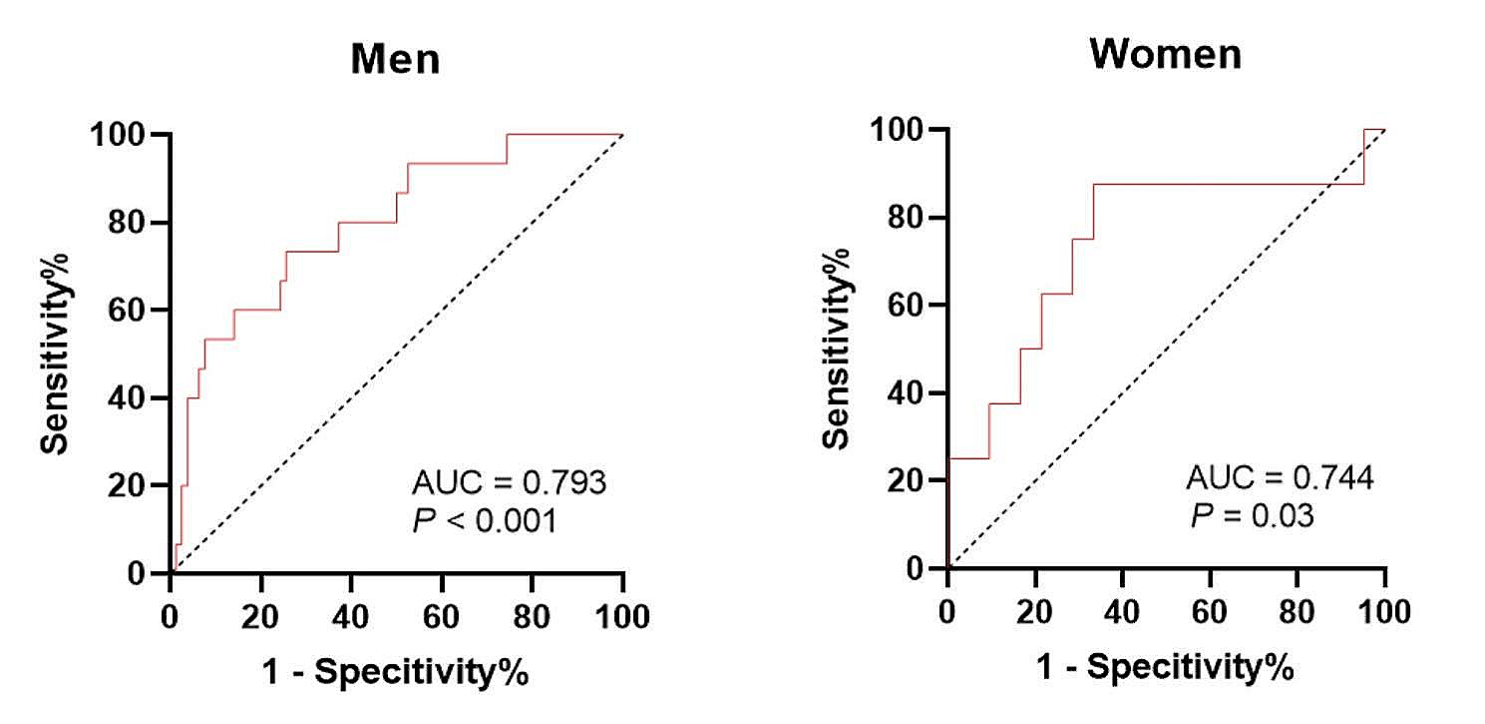
ROC curves of spKt/V in sarcopenia diagnosis. AUC, area under the curve; ROC, receiver operating characteristic

The ROC curves showed the predicted probability of sarcopenia based on spKt/V. The AUC was 0.739 and the best cutoff value of spKt/V on sarcopenia was 1.45 for both sexes with a sensitivity of 63.64% and specificity of 82.64% (Fig. 2; *P* < 0.001). The AUC was 0.793 and the best cutoff value of spKt/V on sarcopenia was 1.33 for men with a sensitivity of 73.33% and specificity of 74.36% (Fig. 3; *P* < 0.001). The AUC was 0.744 and the best cutoff value of spKt/V on sarcopenia was 1.45 for women with a sensitivity of 87.5% and specificity of 66.67% (Fig. 3; *P* = 0.03). In evaluating GNRI with spKt/V, the AUC in the ROC curve analysis was 0.628 (95% CI = 0.48–0.77, *P* = 0.09). The results indicated that spKt/V had a higher predictive capacity in the diagnosis of sarcopenia (Supplementary Fig. S1).

### Associations between spKt/V and Sarcopenia

Models were fitted using multivariate binary logistic regression with sarcopenia as a categorical variable to analyse the associations between spKt/V and sarcopenia, considering the best cutoff value of spKt/V and group by gender. After adjusted for sex, age, diabetes mellitus, dialysis vintage, and BMI, we found that the spKt/V was independently positively associated with sarcopenia (Tables 3 and 4).

**Table 3:** Associations between spKt/V and sarcopenia by binary logistic regression models

Variables	Model 1	*P*	Model 2	*P*
	OR (95% CI)		OR (95% CI)	
Overall				
spKt/V	166.71 (12.05-2307.07)	< 0.001	122.88 (6.18-2442.83)	0.002
Subgroup				
spKt/V < 1.45	Ref		Ref	
spKt/V ≥ 1.45	9.3 (3.43–25.22)	< 0.001	11.75 (3.16–43.67)	< 0.001

OR, odds ratio; CI, confidence interval; spKt/V, single-pool Kt/V_urea_; BMI, body mass index

Model 1: Unadjusted

Model 2: Adjusted for age, sex, diabetes mellitus, dialysis vintage, and BMI

**Table 4:** Association between spKt/V and sarcopenia stratified by gender

Subgroup	Model 1	*P*	Model 2	*P*
	OR (95% CI)		OR (95% CI)	
Men				
spKt/V < 1.33	Ref		Ref	
spKt/V ≥ 1.33	7.76 (2.18–27.63)	0.002	9.73 (2.25–42.11)	0.002
Women				
spKt/V < 1.45	Ref		Ref	
spKt/V ≥ 1.45	14 (1.56-125.26)	0.018	14.52 (1.06-199.67)	0.045

OR, odds ratio; CI, confidence interval; spKt/V, single-pool Kt/V_urea_; BMI, body mass index

Model 1: Unadjusted

Model 2: Adjusted for age, diabetes mellitus, dialysis vintage, and BMI

## Discussion

In this cross-sectional study, we hypothesized that spKt/V is associated with the muscular and nutritional status of patients during thrice-weekly hemodialysis. Primarily, the spKt/V value was found to be a sensitive indicator for sarcopenia in patients on MHD. Furthermore, higher levels of spKt/V were independently associated with a high risk of sarcopenia in MHD patients.

Urea clearance, an indicator of dialysis dose, is expressed as Kt/V [18]. The determination of Kt/V is based on a kinetic model of urea per dialysis, which can be estimated using either a single-pool Kt/V (spKt/V) or a double-pool Kt/V; the latter explains the post-dialysis urea rebound (equilibrium Kt/V [eKt/V]) [19].

The NKF-KDOQI guidelines recommend that for patients with low residual natural renal clearance (KRU, 2 mL/min), the target dialysis dose for thrice-weekly HD is 1.2–1.4 spKt/V per dialysis [6]. It has been shown that spKt/V is a significant predictor of morbidity and mortality in patients receiving HD [20, 21]. Studies have shown a strong correlation between Kt/V and mortality in HD patients [22]. It has been shown that lower than recommended Kt/V (< 1.2) may increase mortality, especially in HD patients among women [23]. In one study, the greatest survival gain of higher HD dose was associated with a Kt/V approaching the 1.6 to 1.8 range [24]. In another study, researchers found that increasing the dialysis dose did not improve mortality in MHD patients, but rather increased the relative risk of death, especially at higher doses ( spKt/V > 1.6) [25]. Therefore, the optimal dialysis dose is still debatable.

Wang et al. found that patients with sarcopenia had higher spKt/V levels than healthy individuals [26]. A study by Kaya et al. found that patients on dialysis with weight and muscle mass loss may experience severe PEW when spKt/V is greater than the target value [8]. Higher Kt/V values are associated with low lean body mass index and a high risk of death in patients on dialysis, ultimately leading to a poor prognosis [27]. However, no study has used dialysis doses to predict skeletal muscle damage in patients.

In patients on MHD, malnutrition is prevalent, and HD survival is associated with the delivered dialysis dose [28]. This may be related to the deleterious effects of malnutrition (manifested as lower V), Which is a well-known risk factor for adverse outcomes and mortality in HD patients [29]. Chertow et al. suggest that patients’ nutritional status should be carefully assessed when spKt/V > 1.6 [7]. Similarly, Owen et al. concluded that mortality in patients on HD was strongly and negatively correlated with dialysis dose, irrespective of whether Kt/V or URR was measured [12], suggesting that increasing the level of dialysis dose is a practical and effective way of reducing mortality and improving clinical outcome [20]. However, the HEMO study by Rocco et al. concluded that dialysis dose interventions were unlikely to have a significant impact on nutritional outcomes [30]. But none of these studies mentioned the effect of a reasonable spKt/V cutoff value on sarcopenia and malnutrition in MHD patients.

In our study, We found that the spKt/V was independently positively associated with sarcopenia in MHD patients, the best cutoff value of spKt/V in sarcopenia was 1.45 for both sexes, 1.33 and 1.45 for men and women, respectively; conversely, in evaluating GNRI with spKt/V, the AUC in the ROC curve analysis was 0.628 (95% CI = 0.48–0.77, *P* = 0.09). These results showed that the cutoff value for women was greater than that for men. In hemodialysis, Kt/V varies between men and women (because of their different body sizes and compositions); Kt depends mainly on the effective clearance of the dialyser and the duration of dialysis [31]. Malgorzata et al. revealed that Kt was similar for males and females and do not depend on V (total body water), which means that the overall capacity of the transport system is similar in females and males, and that the effects of body size and composition are significantly stronger in men than in women [31]. Hence, the body size of men is likely to account for the lower spKt/V than women in this study. Thus the therapeutic target value of spKt/V should be considered for body size and gender.

GNRI is a method of nutritional screening for patients on MHD and an important predictor of mortality in HD patients [32]. However, this study indicated that spKt/V may have a higher optimistic predictive value to identify sarcopenia than the GNRI in these population. Thus, spKt/V may be a clinically useful marker of sarcopenia.

Studies have shown that anthropometry and body composition provide important information about the nutritional status of patients on dialysis, with muscle mass being a marker of protein nutritional status [33]. For example, lean body mass represents the “fat-free” muscle mass in HD patients and is a crucial and useful marker of nutritional assessment in HD patients [34]. Body cell mass constitutes the metabolically active body mass responsible for energy exchange (e.g., muscle mass). Low body cell mass have been identified as nutritional and prognosticator markers [35]. Additionally, ECW and ICW measured using BIS have been introduced as markers of cellular health, and the ECW/ICW ratio has been shown to correlate with malnutrition [36]. And previous studies have found that mCI is a reliable marker of nutritious status in patients receiving HD [37]. Our findings revealed that the lower levels of grip strength, pinch strength, lean body mass, body cell mass, ECW, ICW, BMI, ASMI, and mCI values were related to spKt/V. Higher levels of ECW/ICW values were related to spKt/V. This suggests that there may be more nutrient loss as spKt/V increases. Patients undergoing MHD lose about 1–8 g of protein per hemodialysis, resulting in fewer nutrients to synthesize muscle proteins [38]. Prolonged MHD, therefore, results in patients being exposed to its negative effects, including nutrient loss and increased energy expenditure, which may subsequently lead to malnutrition [39].

Currently, the scientific efforts to reduce morbidity and mortality in patients undergoing HD are focused on three major themes: dialysis dosage, nutrition, and the biocompatibility of dialysis procedures [40]. Adequate assessment and follow-up of the dialysis dose and nutritional status of patients with renal failure may be critical in slowing disease progression and preventing malnutrition. Therefore, spKt/V might be useful as a screening indicator based on medical record information, as an alternative to the Simplified 5-item Rating Questionnaire (SARC-F) scale for estimating sarcopenia versus dystrophy, as described in the consensus [14].

To our knowledge, this was the first study to evaluate the association of spKt/V with sarcopenia in patients on MHD. The present study explored the best cutoff value of spKt/V on sarcopenia, so it can be used as a indicator to identify the patients at risk for developing sarcopenia in advance and malnutrition, according to spKt/V and accordingly initiate interventions to improve the prognosis and quality of life of patients on MHD.

This study had a few limitations. First, the causality could not be determined due to its observational nature of. Second, we cannot exclude the possibility of residual confounders such as inadequate predialysis care. Third, we included patients from the same center, which makes the conclusions less representative. Lastly, we did not evaluate the dietary intake of these patients in a comprehensive nutritional assessment for malnutrition risk. In the future, multicenter studies covering a wider range of confounders should be conducted to establish the association and increase the generalizability of the findings.

## Conclusion

Our study highlighted spKt/V as an independent predictor of sarcopenia in patients on MHD. It demonstrates the importance of spKt/V ≥ 1.45 as an indicator of skeletal muscle wasting. Nutritional status plays an important role in improving the quality of life of patients on dialysis. Therefore, the combination of dialysis dose with muscle mass and nutritional status should be considered as an additional indicator of prognosis in the clinical management of patients on HD. Furthermore, longitudinal studies are needed to further assess the role of dialysis doses on the muscular and nutritional aspects of patients undergoing HD.

### Electronic supplementary material

Below is the link to the electronic supplementary material.

Supplementary Material 1

## Data Availability

No datasets were generated or analysed during the current study.

## Abbreviations

MHD: Maintenance hemodialysis
CKD: Chronic kidney disease
ESRD: End-stage renal disease
PEW: Protein-energy wasting
spKt/V: Single-pool model urea clearance index
NKF-KDOQI: National Kidney Foundation-Kidney Disease Outcomes Quality Initiative
RKF: Residual renal function
URR: Urea reduction rate
BMI: Body mass index
GNRI: Geriatric Nutrition Risk Index
MDRD: Modification of diet in renal disease
eGFR: Estimated glomerularfiltration rate
BIS: Bioimpedance spectroscopy analysis
PhA: Phase angle
ECW: Extracellular water
ICW: Intracellular water
ASM: Appendicular skeletal muscle
ASMI: Appendicular skeletal muscle index
mCI: Modified Creatinine Index
