# Supplementary material for: Single-pool model urea clearance index is associated with sarcopenia and nutritional status in patients undergoing maintenance hemodialysis: a cross-sectional study
Source: BMC Nephrol. 2024 Mar 5;25:80. doi: 10.1186/s12882-024-03510-4 (PMC10916151; doi:10.1186/s12882-024-03510-4)
Supplement: Supplementary file 1 — Supplementary Material 1 [file 12882_2024_3510_MOESM1_ESM.docx]

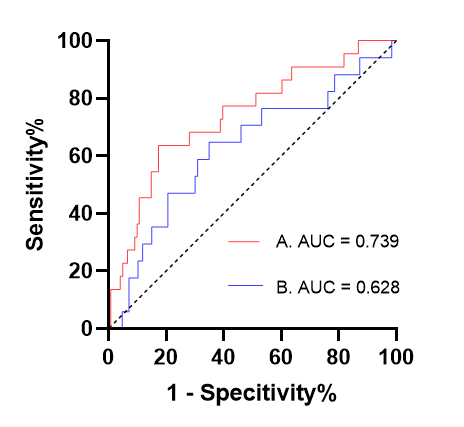


**Supplementary Fig. S1.** ROC curves of spKt/V in sarcopenia (A) and GNRI (B) diagnosis. AUC, area under the curve; ROC, receiver operating characteristic.
